# Diagnosis and management of a rare case of clival chordoma in a young male patient

**DOI:** 10.1016/j.radcr.2024.05.064

**Published:** 2024-06-15

**Authors:** Federica Masino, Manuela Montatore, Marina Balbino, Giuseppe Maria Andrea D'Arma, Gianmichele Muscatella, Rossella Gifuni, Giuseppe Guglielmi

**Affiliations:** a Department of Clinical and Experimental Medicine, Foggia University School of Medicine, Viale L. Pinto 1, 71121, Foggia (FG), Italy; b Radiology Unit, “Dimiccoli” Hospital, Viale Ippocrate 15, 70051, Barletta (BT), Italy; c Radiology Unit, “IRCCS Casa Sollievo della Sofferenza” Hospital, Viale Cappuccini 1, 71013 San Giovanni Rotondo (FG), Italy

**Keywords:** Clival chordoma, Pediatric, Neuroradiology, Oncology, Diagnostic imaging

## Abstract

Chordomas are uncommon bone slow-growing tumors developing from remnants of the notochord. They are typically seen in adults, and rarely in children. We present the case of a 16-year-old male patient with a clival chordoma, presenting with progressive headache and diplopia. In this case report we aim to provide an educational explanation of the radiological findings, diagnostic challenges, and therapeutic and management strategies.

## Introduction

Chordomas are primary bone tumors that originate from embryonic remnants of the notochord. The tumor incidence is about 1% of all intracranial tumors and 4% of all primary bone tumors, with a male-female ratio of 2:1. Their occurrence is more typical in adults (more than 30 years old) but about 5% of cases affect children [1]. with the most frequent localization at the sacrococcygeal level (30%-50%). In 30%-35% of cases, they involve the skull base, most frequently at the spheno-occipital level. These tumors normally grow slowly and cause symptoms related to the mass influence on nearby structures (brainstem, cranial nerves, nasopharynx, spinal cord) [2,3]. Since chordomas arise in bone, they are usually extradural and cause local bone destruction. They are locally aggressive but rarely metastasize (30% of cases) [4]. Metastatic spread of chordomas is observed in 7%-14% of patients and includes nodal, pulmonary, bone, cerebral, or abdominal visceral involvement, predominantly from massive tumors. Due to the local aggressiveness of these tumors, the prognosis is poor with an average 10-year survival of 40% [1].

## Case presentation

**Anamnesis and clinical presentation:** A 16-year-old male patient presented with complaints of persistent headache and double vision. The patient denied having suffered trauma. The patient had had no previous surgery and reported no underlying hereditary or pathological diseases.

Laboratory tests were ordered, all of which were found to be normal. The patient was immediately referred for a neuro-ophthalmological examination which revealed horizontal binocular diplopia. On suspicion of abducens nerve palsy resulting in weakness of the ipsilateral rectus lateralis muscle, an MRI of the brain was requested in order to identify the compressive cause.

**Fig. 1:**
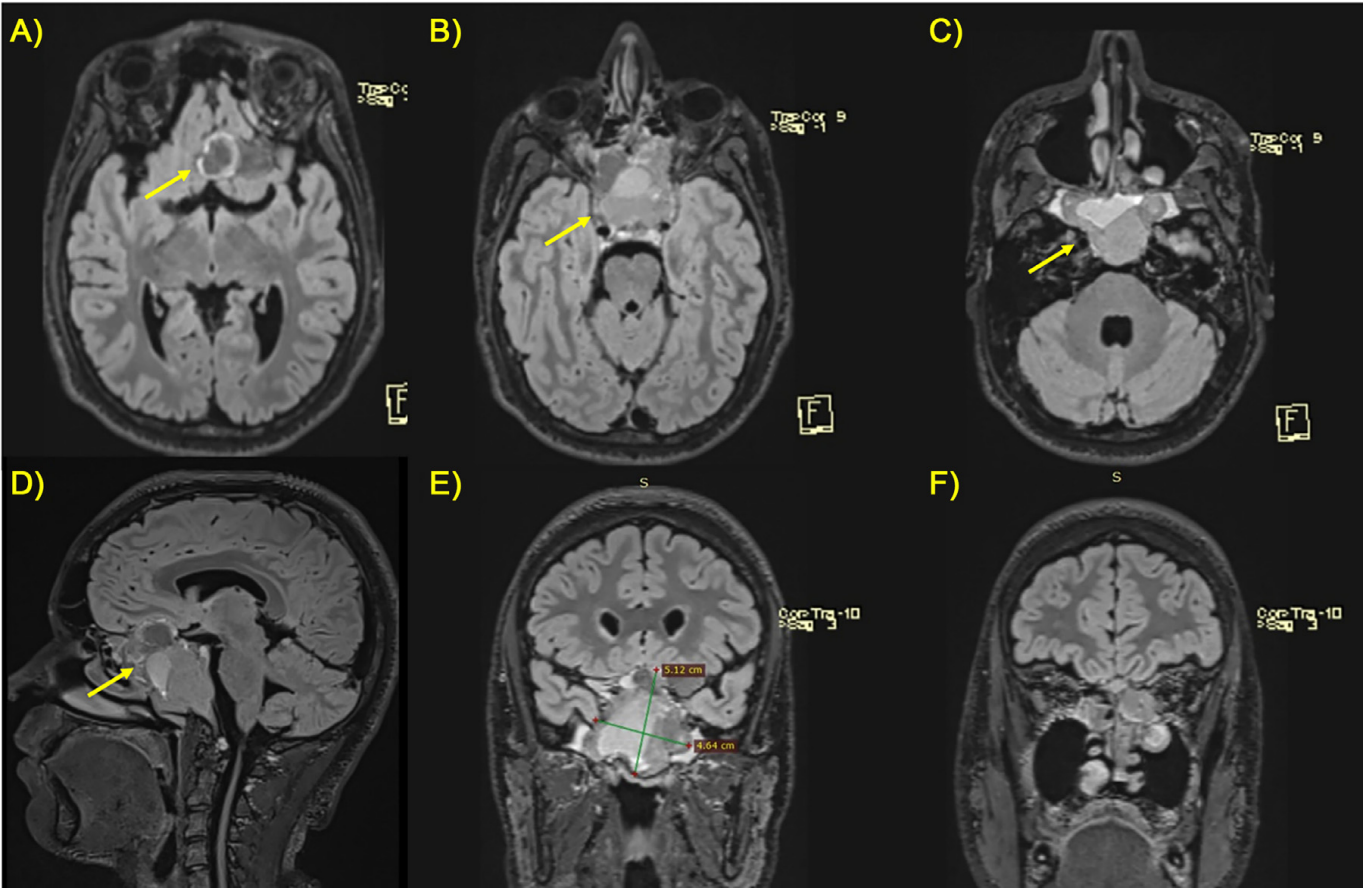
(A-F) FLAIR sequences on 3T MRI. Sequential axial (A-C), sagittal (D) and sequential coronal (E, F) planes showed a voluminous lobulated heterogeneous mass (yellow arrow) originating from the clivus, not recognizable, and extending in the adjacent structures. The mass presented mamelons/vegetations protruding into the nasal cavity, especially on the left side.

**Radiological Findings:** The 3T MRI protocol performed included multiplanar T1, T2, FLAIR, SWI, DWI/ADC, and VIBE sequences after the contrast medium (gadolinium) administration. The MRI examination revealed a large, lobulated mass centered on the clivus, extending into the sellar and suprasellar regions. It measured approximately 5 × 4 cm (craniocaudal x latero-lateral diameter). The mass exhibited heterogeneous signal intensity on FLAIR images, with areas of both hypo- and hyper-intensity (Fig. 1).

**Fig. 2:**
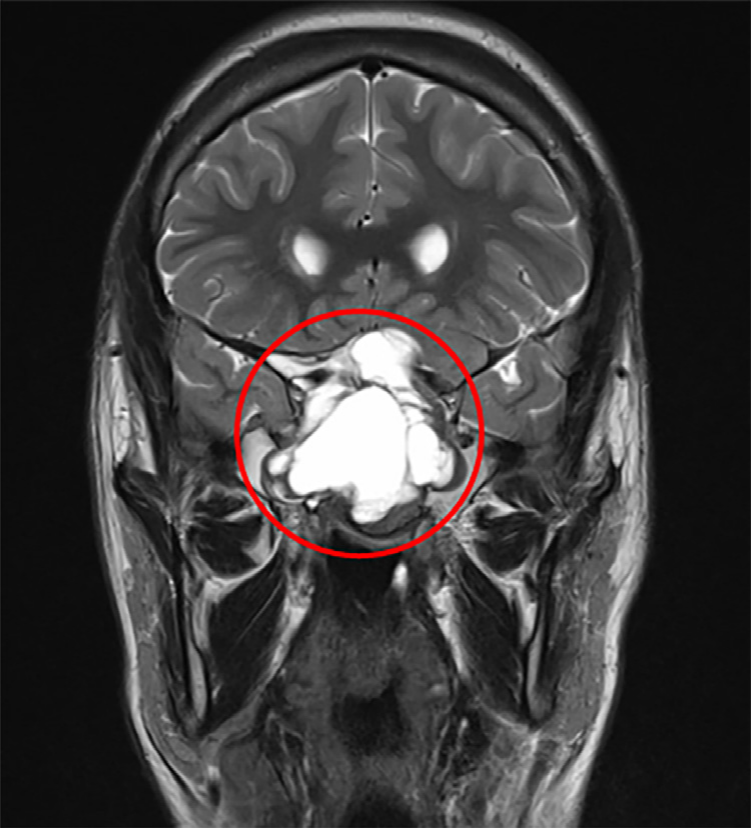
Coronal T2-w sequence on 3T MRI showed hyperintense lobulated mass (red circle) in the clivus with mass effect on the adjacent structures.

T2-weighted images depicted heterogeneous hyperintensity within the lesion, indicating varying degrees of cystic degeneration (Fig. 2).

**Fig. 3:**
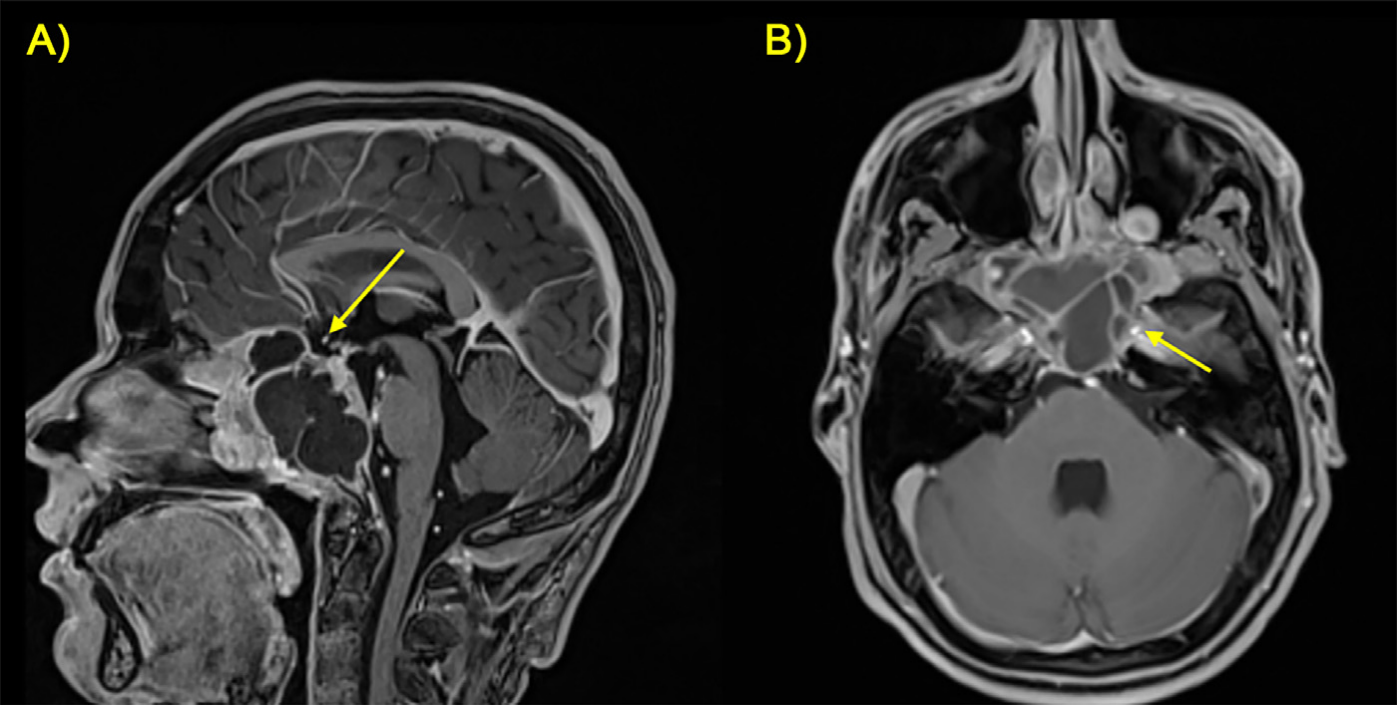
(A, B) Coronal (A) and axial (B) sequences post-contrast administration performed on 3T MRI equipment showed mostly peripherical enhancement of the mass (yellow arrow).

Postcontrast T1-weighted sequences exhibited heterogeneous enhancement throughout the lesion, with areas of central non-enhancement corresponding to necrotic regions (Fig. 3).

**Fig. 4:**
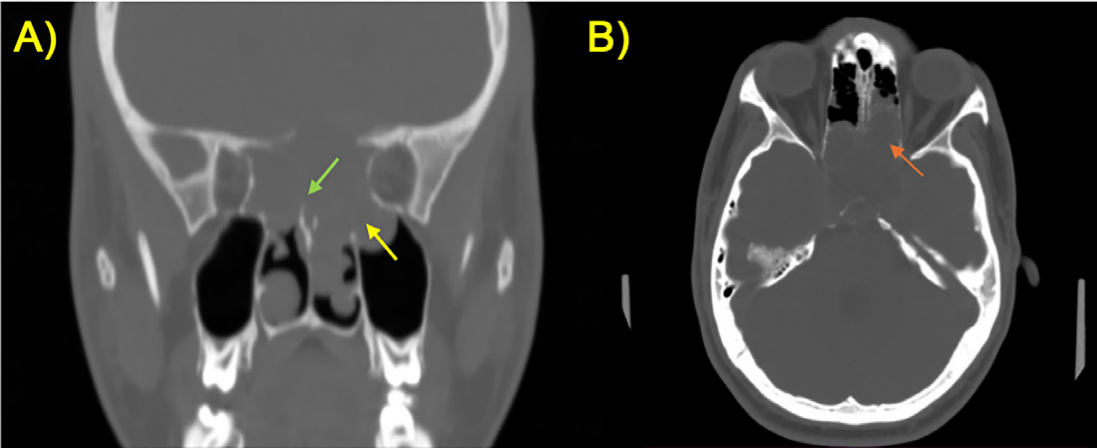
(A, B) CT scan on coronal (A) and axial (B) planes with bone window showed the erosion of the narrowing structures, in particular involving the sellar floor, posterior ethmoid (orange arrow), nasal cavity (green arrow), and left maxillary sinus (yellow arrow).

A maxilla-facial Computed Tomography (CT) was performed to better evaluate the bone structures. It revealed erosion and remodeling of the clivus with soft tissue attenuation, within the lesion, further delineating the extent of bony involvement (Fig. 4).

The patient underwent an additional neck-thorax-abdomen CT after contrast medium administration to exclude the presence of secondaries. No metastases were detected.

**Diagnostic Challenges:** A diagnostic hypothesis of chordoma clivus was raised by the radiologist, but it was not possible to exclude certainly another hypothesis, such as that of chondrosarcoma. Therefore, the rarity of clivus chordomas, particularly in young patients, poses significant diagnostic challenges and since the heterogeneous radiological appearance of chordomas might mimic other skull base lesions, it was necessary for comprehensive imaging evaluation and histopathological confirmation for definitive diagnosis. The patient underwent a trans-nasal endoscopic biopsy of the clival mass, confirming the diagnosis of chordoma histologically.

**Fig. 5:**
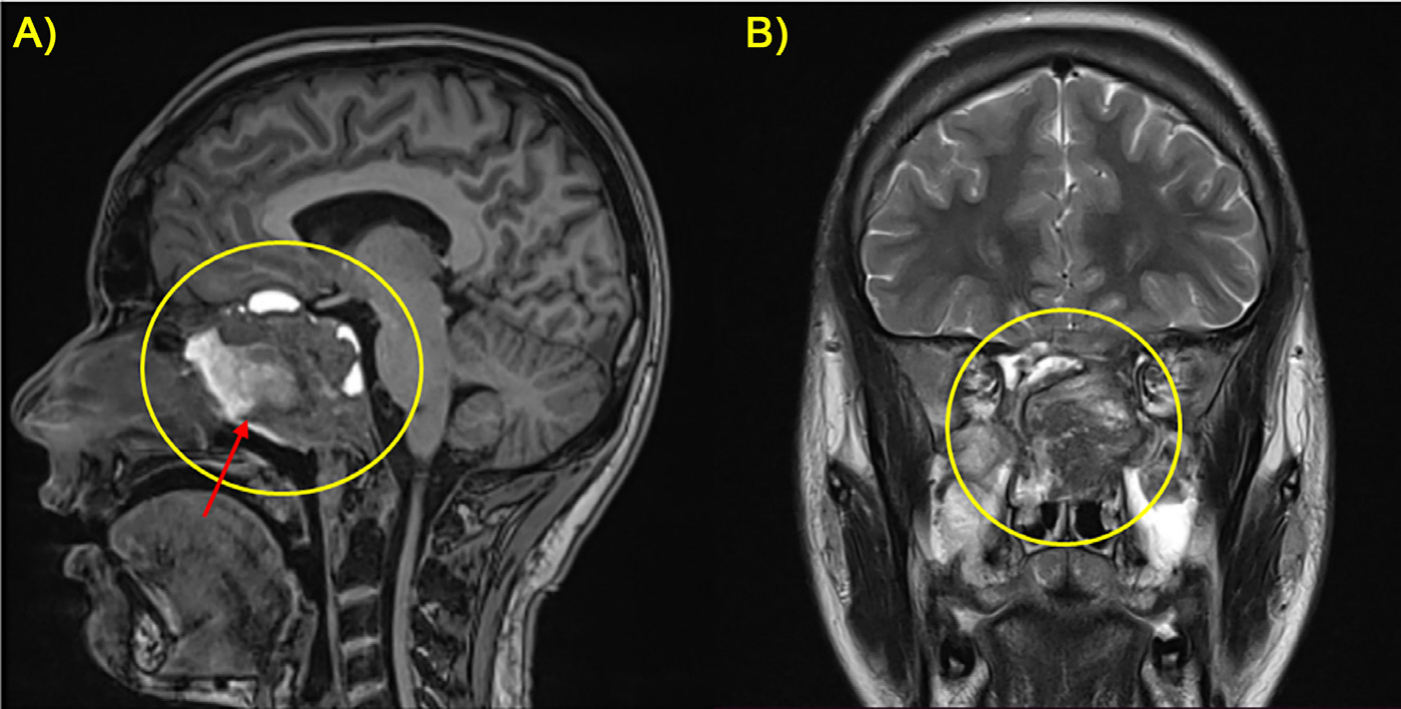
(A, B) Postoperative MRI with sagittal FLAIR and axial T2 sequences showed a voluminous heterogeneous residual mass (yellow circle) with necrotic areas (red arrow).

**Management and Outcome:** Following a multidisciplinary discussion, the patient was scheduled for a staged surgical resection followed by adjuvant radiotherapy to achieve maximal tumor control while preserving neurological function. The surgery's main goal was to achieve sufficient debulking of the tumor and to try resecting it fully. The surgical procedure was performed using an endoscopic endonasal route. The tumor was reached and partially removed since total resection was not achievable due to the very insidious location of the tumor. Post-operatively, the patient's clinical situation improved, and his symptoms subsided. Post-operative MRI performed 1 month after showed signs of surgical intervention on the clivus in addition to residual tumor (Fig. 5).

The patient was sent to an oncologist who agreed on a radiation treatment plan. Actually, his condition remains under control.

## Discussion

The role of imaging is crucial in the evaluation of clival chordoma. The contrast enhanced MRI was the first examination performed, guided by the suspicion of a brainstem tumor and to avoid ionizing radiation since the young age of the patient. In addition, MRI provides excellent anatomical detail, fewer artifacts, and is able to characterize the signal of the lesion usually allowing for a confident preoperative diagnosis.

T1 sequences showed heterogeneous signal intensity, with areas varying from hypo- to hyper- intensity; T2 showed very high signal; DWI/ADC showed areas of diffusive restriction; T1 performed after Gadolinium contrast administration showed heterogeneous enhancement with a honeycomb appearance corresponding to low T1 signal areas within the tumor. Generally, greater enhancement has been associated with poorer prognosis [5,6].

**Fig. 6:**
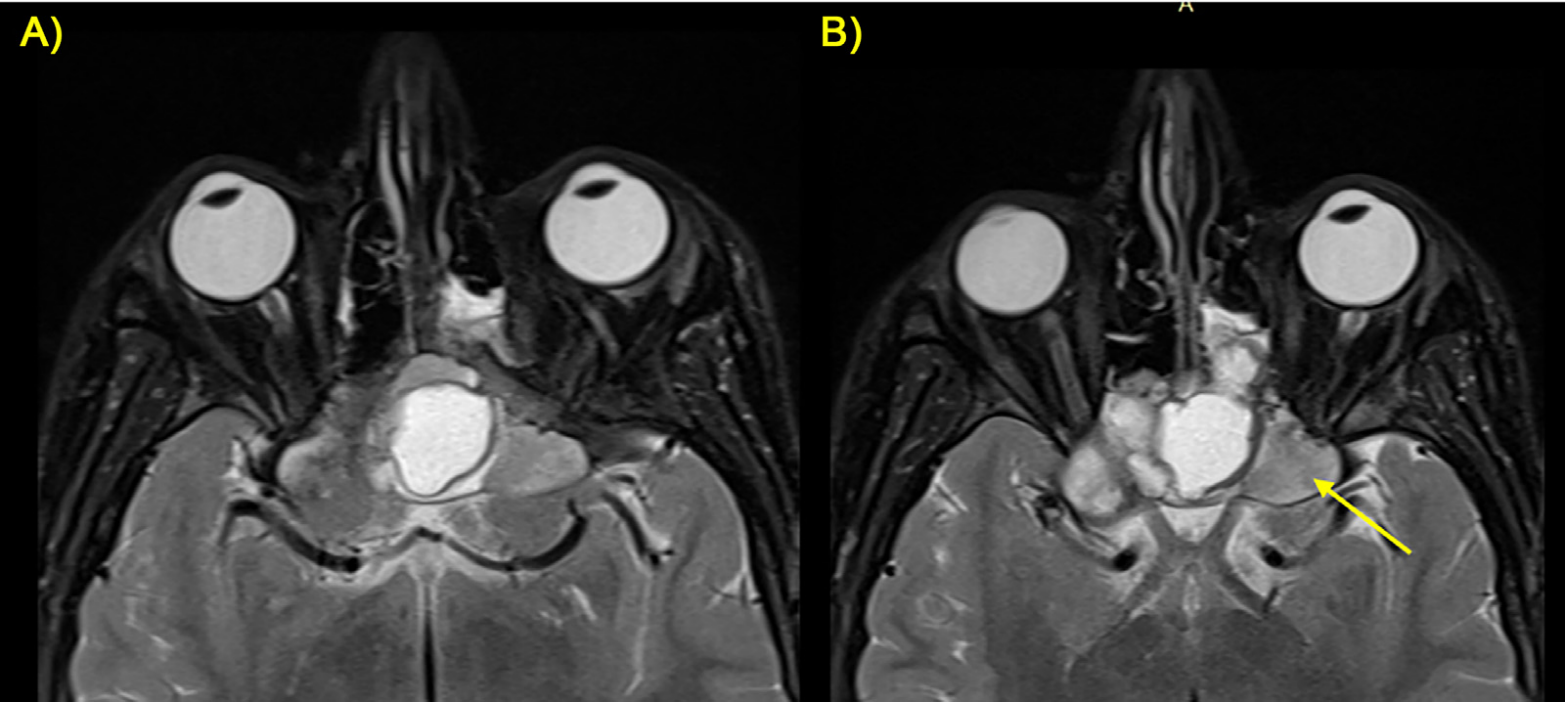
(A, B) Subsequential axial T2-w sequences performed on 3T MRI showed the tumor behavior with compression on the optic chiasm and the left optic nerve (yellow arrow).

The tumor was exerting a mass effect on adjacent structures including the optic chiasm and the left optic nerve (Fig. 6).

MRI is, however, limited in its ability to evaluate calcification and the precise involvement of skull base osteolysis less well than CT, especially for skull base foramina [7,8]. Therefore, CT evaluation was needed to assess the degree of bone involvement and to detect patterns of calcification within the lesion.

**Fig. 7:**
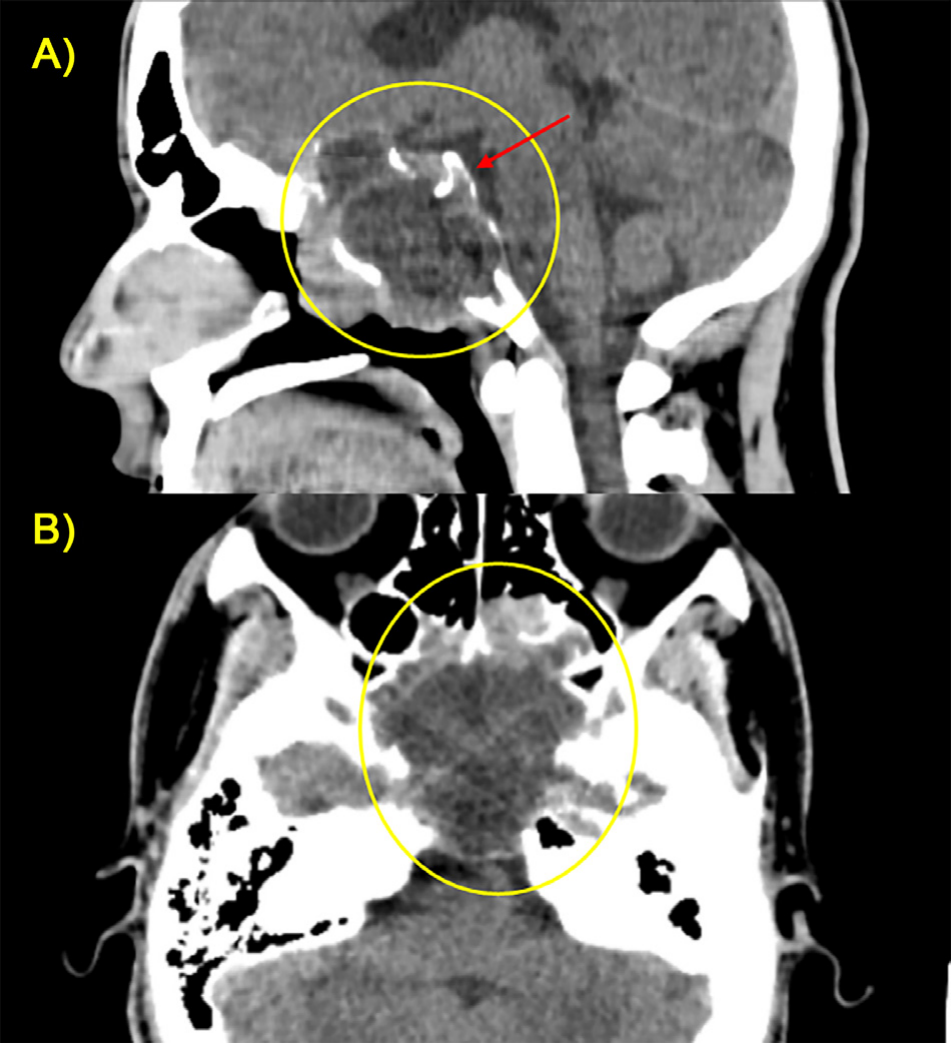
(A, B) Sagittal (A) and axial (B) CT scan without contrast administration showed with the soft tissue window a large heterogeneous mass (yellow circle) with marginal sclerosis (red arrow).

The CT scan showed a lesion with a solid mass with well-circumscribed extension and osteolytic behavior. The mass was very voluminous, and not contained within the bone but showed signs of extra-compartmentalization with erosion of the surrounding bone structures. In fact, the clivus was no longer recognizable. The baseline scan should be evaluated with the soft tissue window (W:350; L: 50) and the bone window (W: 2000; L:500). The soft tissue window applied in the baseline scan showed a heterogeneous density of the tumor, with areas of hypodensity related to necrosis and areas of hyper-density related to hemorrhage. The bone window showed the presence of marginal sclerosis with intra-tumoral calcifications which are mainly related to bone sequestration rather than dystrophic calcifications (Fig. 7).

The postcontrast scan, not carried out in the case under examination as it was already evaluated in the VIBE sequences post Gadolinium in MRI, generally shows a moderate to marked enhancement [5,9].

Since chordoma shows high recurrent rates, follow-up seems obvious. However, there is no accepted evidence-based protocol for monitoring surgically treated clival chordomas [10]. Guler et al. studied the follow-up of chordoma using MRI incorporating DWI and concluded that the detectability of residual chordoma tumor tissue on DWI is better than T2 or FLAIR sequences [11].

When possible, surgical excision has historically been the first course of treatment; in recurrent cases, radiation has been made available. For certain patients, some support the use of both partial or total surgical resection and radiation therapy. As an adjuvant therapy, percutaneous radiofrequency ablation has been tested. Clival chordoma is thought to be among the most challenging tumors to treat because of its anatomical position [9]. Our patient successfully underwent an endoscopic endonasal surgery without any documented complications.

Long-term follow-up is essential for monitoring disease recurrence and treatment-related complications [12]. Recurrence, including seeding along the operative tract, is common [1]. The post-operative control MRI revealed a residual clival chordoma due to the impossibility of performing a complete surgical resection of the tumor.

## Conclusion

Clivus chordomas are exceedingly rare tumors, particularly in young patients. Radiological imaging plays a crucial role in the evaluation of these lesions, aiding in accurate diagnosis and treatment planning. A multidisciplinary approach involving neurosurgery, radiology, and oncology is essential for optimal management and long-term outcomes in patients with clival chordoma.

## Patient consent

Informed consent was obtained from the patient.
